# Novel approach to providing child and adolescent mental health education to allied health services

**DOI:** 10.1192/bjo.2021.389

**Published:** 2021-06-18

**Authors:** Michael Foster, Sally Arnold

**Affiliations:** 1 The Darwin Centre; 2 Harplands Hospital

## Abstract

**Aims:**

To create and deliver a positive educational session for allied health services on prominent child and adolescent mental health conditions. It was hypothesised that delivering tailored teaching sessions on a range of child and adolescent mental health conditions would help improve the knowledge of allied health services. A quiz was administered at the beginning and end to assess the effectiveness of the sessions.

**Background:**

In early 2019, a request was made by Staffordshire youth drug and alcohol service for an informal teaching session on prominent mental health conditions experienced by those under 18. The team often encountered the requested conditions but had no role in managing or treating them resulting in weaknesses in their knowledge. There was a strong desire to learn more about what the cause, presentation, diagnosis and management was of these too. An interactive, 60-75 minute session was requested on ADHD, autism, depression, anxiety, emerging emotionally unstable personality disorder, bipolar affective disorder, and schizophrenia.

**Method:**

Sessions were conducted at the local drug and alcohol service, and at 2 regional social services, in autumn 2019. A 21 question quiz, 3 questions on each topic, was taken at the start and end of each session. The quiz content was covered within the teaching session, as well as time for questions, then marked and converted into a percentage.

**Result:**

19 quizzes were taken; either by individuals or within pairs. The average score before the teaching was 43%, increasing to an average of 90% after the teaching. The quiz showed good knowledge on anxiety and depression before the teaching, with an average pre-test score of 66%, whereas knowledge on the other topics was less. Post-test scores increased to 100% for most areas, but scores for ASD and bipolar were both 66%.

**Conclusion:**

Feedback from the sessions was positive and staff across both services demonstrated a significant improvement in their understanding of prominent CAMHS mental health conditions. Further education and a change of approach to teaching is required for autism and bipolar affective disorder, both of which are challenging and broad topics.

The pre-teaching results do however demonstrate there is a need for better inter-agency education within teams, as well as reciprocal teaching so that knowledge from different teams can be shared. Further sessions are being proposed for other social services and general practises.

